# Editorial: Advances in targeted delivery of natural flavonoids

**DOI:** 10.3389/fnut.2023.1266509

**Published:** 2023-08-24

**Authors:** Yang Xu, Zhenquan Jia, Hongming Su

**Affiliations:** ^1^Ningbo Innovation Center, Zhejiang University, Ningbo, China; ^2^Department of Food Science and Nutrition, Zhejiang University, Hangzhou, China; ^3^Department of Biology, University of North Carolina at Greensboro, Greensboro, NC, United States; ^4^Department of Food Science and Nutrition, University of Minnesota-Twin Cities, Saint Paul, MN, United States

**Keywords:** flavonoids, inflammation, gut microbiota, obesity, oxidative stress

Flavonoids are a diverse group of phytochemicals widely found in fruits and vegetables. The main dietary sources of flavonoids include berry fruits, tea, citrus, apples, and colored grain. Accumulating evidence indicates that flavonoids have health-promoting effects, including anti-inflammation, anti-oxidative stress, regulation of lipid and glucose metabolism disturbances, and anti-aging (1–3). To elucidate the health-promoting effects of natural flavonoids, it is important to understand their concentration and forms in plasma and tissues following dietary intake. However, the bioavailability, absorption, and metabolism mechanism of natural flavonoids in the body are complex and need further study. Furthermore, emerging evidence suggests that dietary flavonoids have limited accessibility and bioavailability in the gastrointestinal tract diminishing their potential health benefits. Therefore, exploring targeted delivery methods such as nanoparticles or self-assembly is crucial to enhance the bioavailability and bioactivity of natural flavonoids.

This Research Topic aims to provide the latest insights into the health-promoting effect of flavonoids against inflammation, oxidative stress, and lipid and glucose metabolism disorders. In addition, this topic also seeks to reveal the recent research progress on the bioavailability, absorption, and metabolism mechanisms of natural flavonoids and to develop targeted delivery systems to improve the bioavailability and biological activity of natural flavonoids.

Four research papers in this Research Topic cover the aforementioned aspects, and Figure 1 presents the graphical abstract of this Research Topic. Two papers highlight the potential health benefits of cyanidin-3-O-glucoside (C3G). Wang et al. showed that 40 mg/kg C3G, extracted from Chinese bayberry, can alleviate antibiotic-associated diarrhea by modulating gut microbiota and down-regulating inflammatory factors in the NF-κB pathway. The researchers identified the specific bacterial strains affected by the C3G treatment, and the authors noted that the compound had a protective effect on the intestinal barrier. The mechanism suggests that C3G restored gut microbiota composition by reducing the abundance of harmful bacteria and increasing the relative abundance of beneficial bacteria. Furthermore, C3G reduced inflammation, reestablished expression of intestinal tight junction proteins (claudin-1 and ZO-1), and improved intestinal permeability. Overall, this study suggests that C3G holds promise as a natural treatment for antibiotic-associated diarrhea.

**Figure 1:**
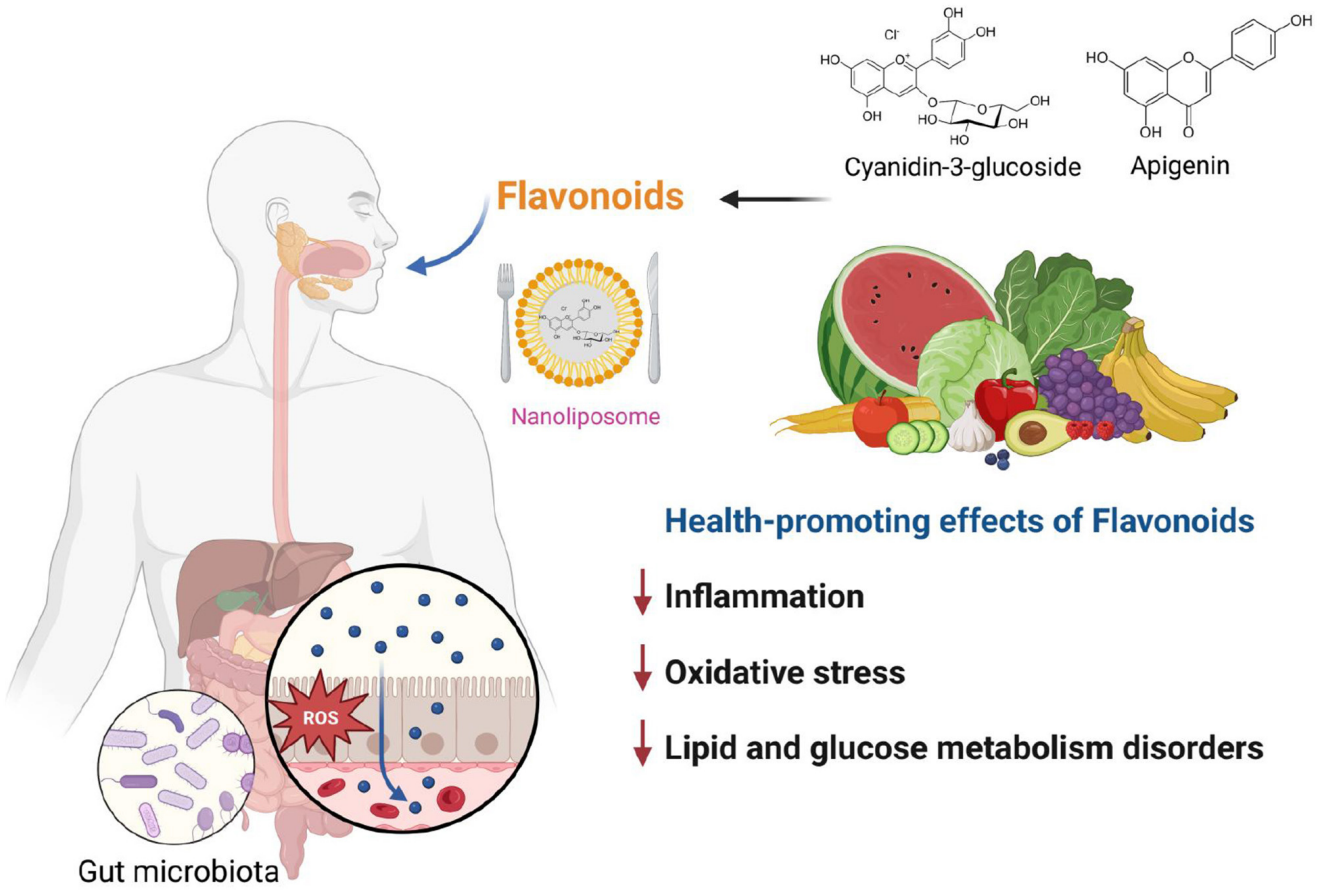
Graphical abstract of the Research Topic. This figure was created using BioRender.com.

Accumulating evidence indicated that C3G has limited absorption efficiency and efficacy in the human body due to its low stability and bioavailability (4). Therefore, strategies to improve bioavailability are of great importance for exploring the absorption mechanisms and functional applications of C3G. Nanoliposomes have been reported to improve the transport efficiency and uptake rate of C3G, but the underlying mechanism remains unclear. In this Research Topic, Yang et al. showed that the uptake and transport of C3G nanoliposomes were concentration-dependent and affected by temperature (37 and 4°C). Using endocytic inhibitors in a Caco-2/RAW 264.7 co-culture model, this study revealed that C3G nanoliposomes enter cells via endocytosis (Yang et al.). Furthermore, C3G nanoliposomes significantly decreased the expression of pro-inflammatory cytokines (TNF-α, IL-1, IL-6, and IL-8) compared with C3G, suggesting a better anti-inflammatory potential. Macropinocytosis and endocytosis mediated by carrier protein (clathrin) are mainly involved in the uptake of C3G nanoliposomes. C3G nanoliposomes may be more effective in treating intestinal inflammatory illnesses caused by lipopolysaccharide. In conclusion, this study provides valuable insights into the cellular uptake, transport mechanism, and anti-inflammatory effect of C3G nanoliposomes, which may have potential applications in the development of functional foods to prevent and treat intestinal inflammation.

The other two papers focused on the biological activity of flavonoids, specifically apigenin and goji leaf flavonoids. Apigenin is a naturally occurring flavonoid found in various vegetables and fruits. Fu et al. found that apigenin significantly attenuated the pathological damage to the intestinal lining, increased the number of goblet cells and mucin secretion, elevated the expression of the anti-inflammatory cytokine IL-10, and inhibited the expression of the pro-inflammatory cytokines TNF-α, IL-1, IL-6, and MPO activity in colon tissue. Apigenin stimulated the expression of ZO-1, claudin-1, and occluding, contributing to the repair of the intestinal barrier. In addition, apigenin altered the disorganized gut microbiota by controlling the abundance of bacteria such Akkermansia, Turicibacter, Klebsiella, and Romboutsia as well as their metabolites (SCFAs) thereby attenuating the damage to the colon caused by dextran sodium sulfate (DSS). Overall, this study suggests that apigenin has potential as a natural and safe therapeutic agent for the prevention or treatment of ulcerative colitis.

Liao et al. investigated the potential benefits of flavonoids from *Lycium barbarum* leaves in reducing obesity in high-fat diet-fed mice. This study found that these flavonoids can effectively reduce body weight, improve glucose and lipid metabolism, and modulate gut microbiota composition in obese mice. The results suggest that *Lycium barbarum* leaves could serve as a promising natural source of flavonoids for preventing and treating obesity-related metabolic disorders.

Taken together, the above findings provide compelling evidence of the health benefits of flavonoids. This Research Topic would provide a deeper understanding of dietary flavonoids in the regulation of against inflammation, oxidative stress, lipid and glucose metabolism disorders, and homeostasis of gut microbiota. It is also important to further explore other potential health benefits of dietary flavonoids and to develop targeted delivery systems that increase the bioavailability and biological activity of natural flavonoids.

## Author contributions

YX: Writing—original draft, Writing—review and editing. ZJ: Writing—original draft, Writing—review and editing. HS: Writing—original draft, Writing—review and editing.
